# Noise Amplification in Human Tumor Suppression following Gamma Irradiation

**DOI:** 10.1371/journal.pone.0022487

**Published:** 2011-08-05

**Authors:** Bo Liu, Shiwei Yan, Xingfa Gao

**Affiliations:** 1 Key Laboratory of Beam Technology and Material Modification of Ministry of Education, College of Nuclear Science and Technology, Beijing Normal University, Beijing, China; 2 Key Laboratory for Biomedical Effects of Nanomaterials and Nanosafety, Institute of High Energy Physics, Chinese Academy of Sciences, Beijing, China; 3 Beijing Radiation Center, Beijing, China; University of Maribor, Slovenia

## Abstract

The influence of noise on oscillatory motion is a subject of permanent interest, both for fundamental and practical reasons. Cells respond properly to external stimuli by using noisy systems. We have clarified the effect of intrinsic noise on the dynamics in the human cancer cells following gamma irradiation. It is shown that the large amplification and increasing mutual information with delay are due to coherence resonance. Furthermore, frequency domain analysis is used to study the mechanisms.

## Introduction

How cells process noise is a challenging problem in illuminating the principle of intracellular motifs [1]–[3]. Shen-Orr *et al.* [4] find that much of a biological network is composed of repeated appearances of several highly significant motifs. Some network motifs have been used recently to explore the principle of cellular systems [4]–[6]. In two well-studied examples, the p53-Mdm2 regulatory network and the NF-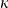
B signaling pathway, noisy oscillations in the cells following activation signals were studied in the experimental [7]–[12] and theoretical [13]–[31] aspects. The core circuit consists of one of the most common network designs, a negative feedback loop [32], [33], where the active transcription factor promotes the transcription of its own repressor.

Mathematical models have achieved oscillatory dynamics by introducing *ad hoc* time delays to reproduce those that a system incurs when the various molecular components are manufactured [21], [22], [24], [25], [28], [30], [34]–[39]. Related works have been performed on many fields of research, where delays were found to play a central role. For example, the importance of delay has also recently been recognized in neuronal dynamics [40]–[43]. From the mathematical point of view, the difference between single-cell experiments and cell population experiments of simple regulatory networks arises from stochastic events in individual cells that are averaged out in cell population. As the noise intensity of the regulating species increases, the noise intensity of the regulated one also appears to increase. Noise can induce many phenomena in nonlinear dynamical systems, including stochastic resonance, coherence resonance, pattern formation and so on. Lots of original research [44]–[49] and review [50]–[52] articles have been devoted to the stochastic resonance phenomenon. Noise-induced patterns in semiconductor nanostructures have been recently investigated by means of theoretical models [53], where random fluctuations play an essential role. Our presented results are crucially relying on coherence resonance, which has been recently studied for temporal systems [54]–[57] and spatially extended systems [58]–[63]. Specifically the relevance of intrinsic noise was elaborated on periodic calcium waves in coupled cells [64] and spatial coherence resonance in excitable biochemical media [65] induced by internal noise. A recent comprehensive review [66] has been done on the stochastic coherence. The large amplification results from the existence of coherence resonance with delay and noise.

In this article, by exploiting a microscopical signal-response model which was proposed in our previous articles [37], [38] for studying the dynamical mechanism of the oscillatory behaviors for the activities of p53 and Mdm2 proteins in individual cells, we will explore the mechanism of noise amplification by considering the stochastic events in the cells.

## Results and Discussion

### Noise amplification

We introduce the probability 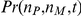 for the p53 and Mdm2 populations 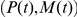 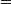 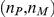. Then the master equation for 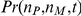 is given by
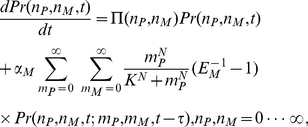
(1)where 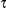 is added to account for the time delay between the activation of p53 and the induction of Mdm2. 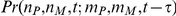 is the joint probability distribution of having 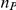 p53 molecules, 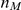 Mdm2 molecules at time 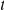 and 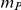 p53 molecules, 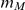 Mdm2 molecules at time 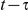. 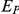 and 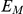 are the unitary shift operators,
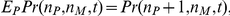


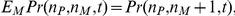
and
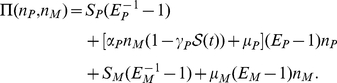
(2)

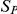
, 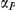, 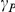, 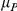, 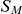, 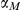, 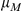, 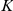, 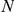 and 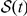 are the parameters denoting various mechanisms as represented in our previous papers [37], [38].

Assume that the time delay 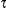 compared with other characteristic times of the system is large, so the processes at time 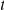 and 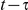 are weakly correlated as 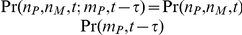. Adopting this approximation, we get
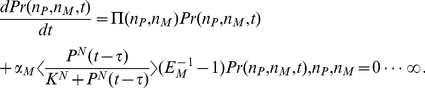
(3)


The generating function 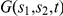 is defined as
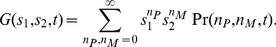
(4)We convert the infinite set of ordinary differential equations (3) to a single partial differential equation for 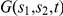,
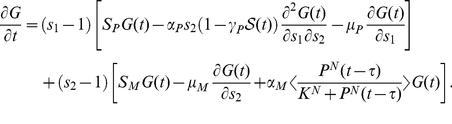
(5)


The moments of the probability distribution can be found by expanding the generating function near 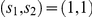,
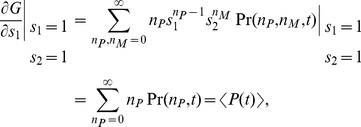
(6)

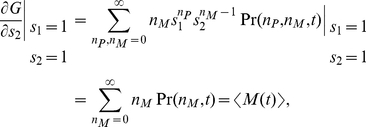
(7)

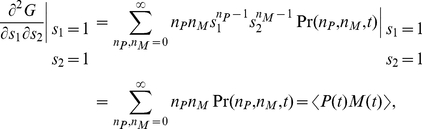
(8)

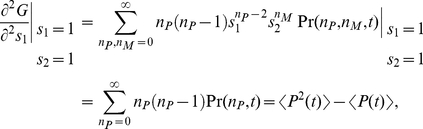
(9)

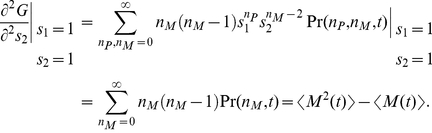
(10)


**Figure 1:**
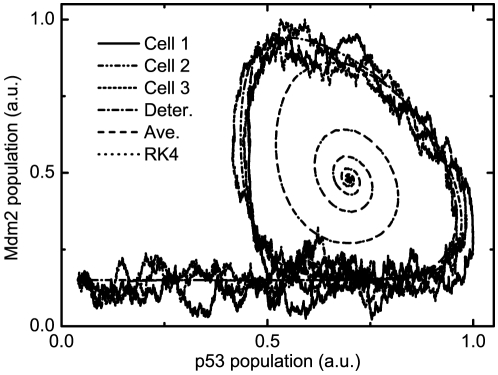
Normalized phase plot 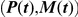 in 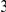 individual MCF7 cells following gamma irradiation, deterministic (Deter.) solutions 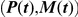, and average (Ave.) populations 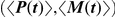 in population of cells obtained with the exact DSSA (Ave.) and fourth-order Runge-Kutta (RK4) solutions of Eqs. (12) where the numerical values of 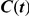 and 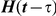 are obtained with the exact DSSA. The parameters are chosen as 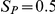 min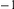, 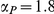 min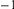, 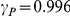, 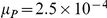 min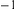, 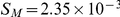 min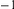, 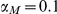 min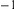, 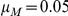 min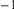, 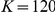, 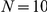, 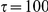 min and 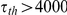 min.

Substituting the expansion
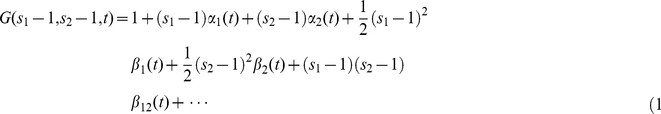
(11)into Eq. (5) we obtain
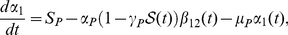
(12a)

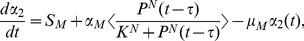
(12b)where the functions 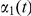, 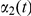 and 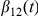 are Eqs. (6), (7) and (8), respectively. Above is the presentation of the derivation by help of generating functions. In fact, it delivers the same moment equations as the derivation by averaging the master equation. Both approaches run finally into equivalent approximations and problems if decoupling the moments. By the comparison between Eqs. (12) and the corresponding deterministic equations described in our previous papers [37], [38], we find that due to
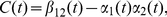
(13a)

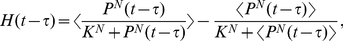
(13b)the limit cycle of the deterministic description [37], [38] changes to a decaying scheme as shown in Fig. 1.

From our numerical results, the reason for the decaying can be considered as dephasing that is mainly caused by differences in the Hill function 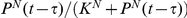 between the cells. The reason that Hill functions are different is the different states of the different cells at time 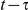, i.e., some dephasing happened at time 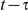 for it to have this impact. The delay further amplifies the differences between cells, causing further dephasing. but if we take two cells with identical state space paths, their Hill functions will also be the same.

This initial difference between the particle numbers of chemical species in different cells, which causes the difference in Hill function at later time, is entirely caused by the intrinsic noise. In fact, any oscillating chemical system, with or without delayed dynamics, will demonstrate dephasing between different realizations, and it isn't an artifact of the delayed dynamics themselves, although this will undoubtedly cause further decorrelation of different realizations at later time, which causes the damped behavior at the population level (which can be thought of as simply taking a large number of realizations of the same stochastic system). Essentially, the value which the cell population converges to is simply approximately the mean of the invariant distribution of the chemical species for one cell, multiplied by the number of cells in the population of interest. This can be shown more rigorously for large populations using the ergodic property of the system.

**Figure 2:**
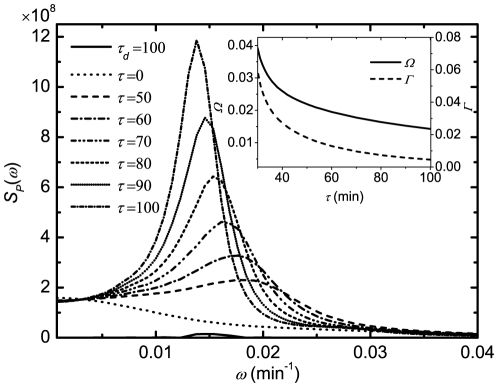
A plot of the average power spectrum 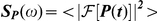 as a function of frequency 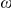 with (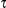 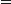 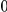,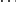,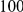 min) and without (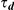 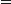 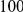 min) noise, where 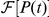 is Fourier transform of p53 dynamics from the time to the frequency domain, and the p53 dynamics 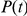 is obtained with the DSSA. Inset: 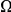 and 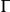 fitted with Eq. (14) vs. 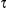. The other parameters are as in Fig. 1.

Fig. 2 shows the average power spectrum 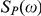 for 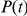 time series as a function of frequency 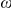. We also plot the spectrum of the corresponding deterministic model, with delay (e.g., time delay 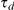 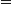 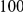 min in Fig. 2) but without noise, to compare its spectrum with stochastic ones. It can be clearly seen that 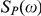 without noise is much smaller than those with noise. Significantly, for the cases with large 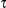 (especially, 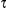 is larger than the Hopf bifurcation point 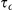), there are obvious peaks appearing in 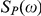 for 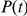 at 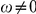. This tells us that there is a very large amplification of intrinsic noise due to the resonant effects. This characteristic phenomenon may be termed as coherence resonance with delay and noise, for distinguishing from the “ stochastic resonance” in common sense.

The peak frequency corresponds to the characteristic frequency of the solution of Eqs. (12), which represents the mean frequency of Fourier transform 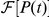. It is very intriguing that the width of 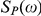 represents the dephasing effects, which gives the damping strength on the amplitude of 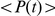. In order to analyze this resonant oscillation more transparently, we phenomenologically fit 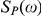 for the cases with large 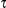 (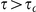) shown in Fig. 2 by a formula
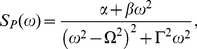
(14)where the parameters 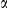, 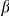, 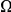 and 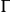 are 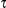-dependent. Note that Eq. (14) can be analytically derived with the chemical Langevin equations corresponding to Eqs. (1) under the linearization approximation.

The resultant 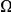 and 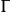 are shown in the inset picture of Fig. 2. It is obvious that the mean frequency 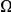 decreases against 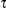, which is consistent with the conclusion described in our previous article [37]. This is particularly important in biology because in general the low frequency is much more significant than higher frequency in biological systems. 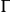 also decreases as 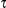 increasing, which means that the oscillation may dominate the evolution of 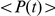 and lasts for rather longer time for very large 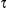. This phenomenon is very intriguing from the biological point of view because it may tell us that the time delay induced by the underlying multistage reactions may weaken the effects of stochasticity and strengthen the oscillation of the relevant molecules.

Mutual information (MI) is meaningful to discuss resonant phenomena [67], so we give the mutual information between the two components p53 and Mdm2 in the nonlinear delayed-feedback network motif. MI is a measure of the amount of information that one random variable interacts with another. It is the reduction in the uncertainty of one random variable due to the knowledge of the other [68]. MI between variables 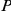 and 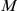 can be represented as
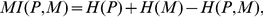
(15)where the Shannon entropy, 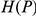, 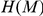, and the joint entropy 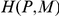 are defined as
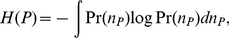
(16a)

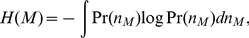
(16b)

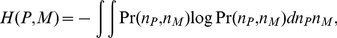
(16c)where 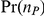 and 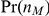 are margin distribution functions and 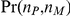 is the joint distribution function. Thus the MI can be represented as
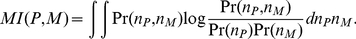
(17)


**Figure 3:**
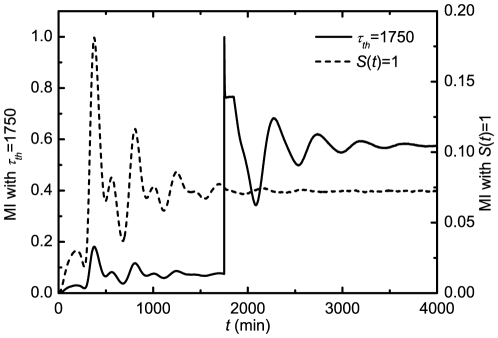
Evolution of Mutual information (MI) with 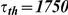 min and 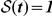. The other parameters are as in Fig. 1.

**Figure 4:**
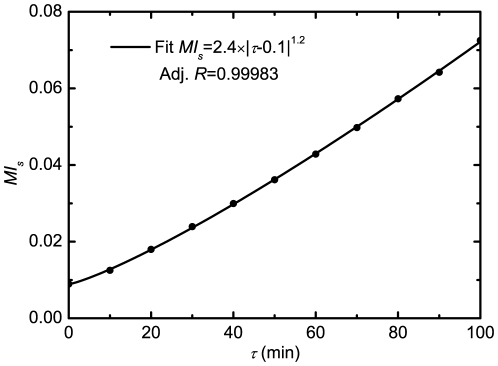
Mutual information (MI) in steady state as a function of time delay 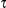, where 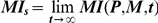. The fit function and its adjusted 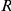-Square are indicated. The parameters are as in Fig. 1.

MI is zero if and only if the two random variables are strictly independent [69]. Numerically calculating the mutual information between trajectories is in general a formidable task [70], since the joint distribution of continuous variable is smoothly obtained only for large scale stochastic simulation. Intensive work has been done on estimating the mutual information. Khan *et al.* [71] reviewed three MI estimators: Kernel density estimators, k-nearest neighbor method and Edgeworth expansion. Recently, Suzuki *et al.* [72] proposed a novel MI estimator called Least-Squares Mutual Information, and discussed the characteristics of the three existing approaches. However, it is accessible here due to the discreteness of the system with the exact delay stochastic simulation algorithm (DSSA) [73]. Information theory [74] provides a natural framework for many problems in biological information processing. The Shannon mutual information has been applied to study the stochastic resonance (SR) [67], [75], [76], instead of the signal-to-noise ratio (SNR). It can be seen from Fig. 3 that when the DNA is damaged, the phosphorylation of p53 modifies its binding properties to Mdm2, so MI is small; But when the signal is completely resolved, e.g., after 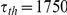 min, MI is large because the amount of p53 is kept low and tightly regulated by the genetic network of Mdm2 and p53 itself. Fig. 4 shows that MI in steady state increases with the increase of time delay due to the coherence resonance.

### Fourier analysis

To describe the nonlinear dynamics more clearly, we use frequency domain analysis method to study the mechanisms of the p53 network motif. Our model can be described by a set of chemical Langevin equations corresponding to Eqs. (1),
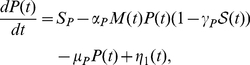
(18a)

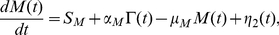
(18b)where 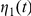 and 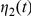 are Gaussian white noise, 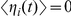, 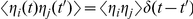, 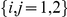 [77].

In order to analyze our model in the frequency domain, we first replace 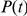 and 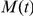 in Eqs. (18) by
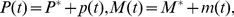
(19)where 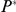 and 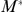 represent the stationary solutions of the deterministic equations of Eqs. (18) with 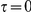, which satisfy the equations
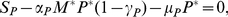
(20a)

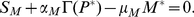
(20b)


Since we are discussing the solution in the oscillatory scheme, here the signal 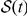 is set to be 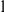. If one hopes to discuss the case of the stationary solution in 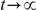, he can simply set the parameter 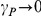 mathematically, because at 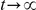, the damage can be supposed to be completely resolved as 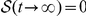, i.e., the signal 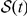 is first set to 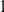, later 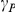 is removed because 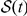 is becoming 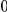 if time tends to infinity. Then Eqs. (18) can be rewritten as
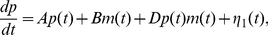
(21a)

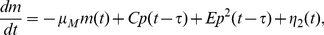
(21b)where
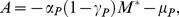
(22a)

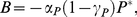
(22b)

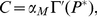
(22c)

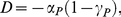
(22d)

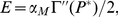
(22e)and the nonlinear term is kept up to the second order in 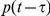.

The Fourier transformations of Eqs. (18) take the form
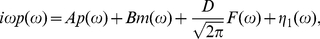
(23a)

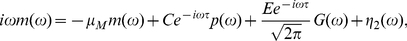
(23b)where
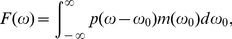
(24a)

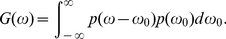
(24b)


Since Eqs. (23) are integral equations, they can be solved by interpolation method and truncated at a specific order, the following calculation includes convolutions in the spectral presentation replacing the nonlinear items in the temporal one and truncating them, e.g., we can first solve the linear equation
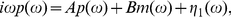
(25a)

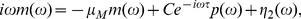
(25b)substitute the solutions 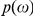 and 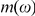 of Eqs. (25) into Eqs. (24), and then 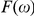 and 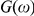 are functions of 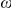. Under the approximations of weak noise and weak negative feedback mechanism, in this paper, the solutions of both 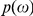 and 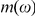 are retained up to the second order of 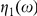 and 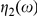, because for Gaussian noise, the terms of higher order can be omitted in Ito-Wiener approximation. The validation of such approximations will be discussed with our numerical simulation later. We define
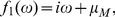
(26a)

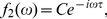
(26b)

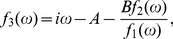
(26c)

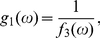
(27a)

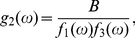
(27b)

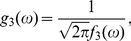
(27c)

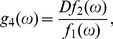
(27d)

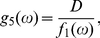
(27e)

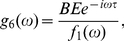
(27f)

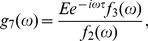
(27g)and then it can be derived from Eqs. (23) that
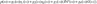
(28a)

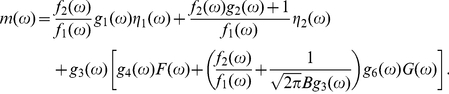
(28b)


By defining the intermediate variables,
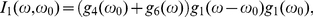
(29a)

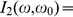
(29b)

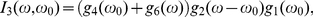
(29c)

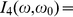
(29d)

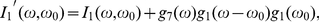
(30a)

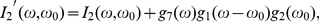
(30b)

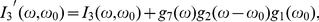
(30c)

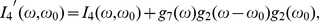
(30d)Eqs. (28) can be written as
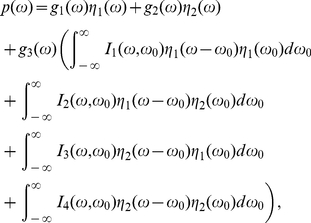
(31a)

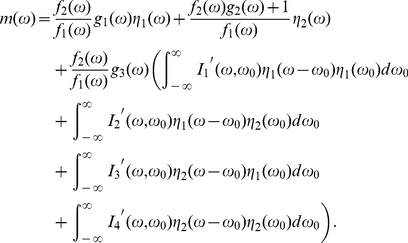
(31b)Let
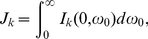
(32a)

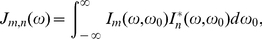
(32b)

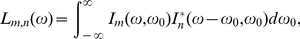
(32c)

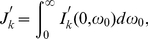
(33a)

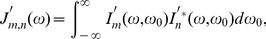
(33b)

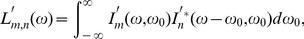
(33c)where 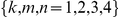. Then the correlation functions of 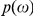 and 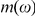 can be expressed as
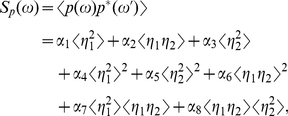
(34a)

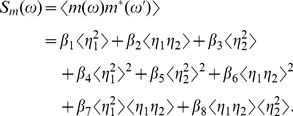
(34b)


The parameters 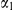, 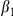, 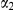, 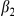, 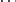, 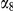, 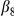 represent the contributions of 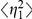, 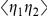, 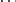, 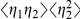 to the correlation functions 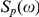 and 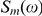, respectively. With the aid of the intermediate variables, those parameters can be expressed as
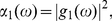
(35)

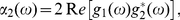
(36)

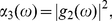
(37)

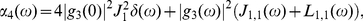
(38)

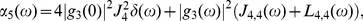
(39)

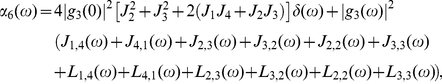
(40)

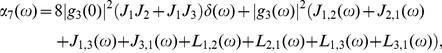
(41)

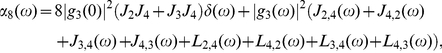
(42)

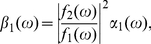
(43)

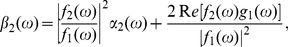
(44)

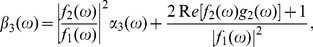
(45)

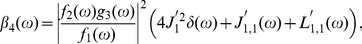
(46)

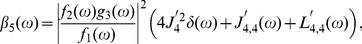
(47)

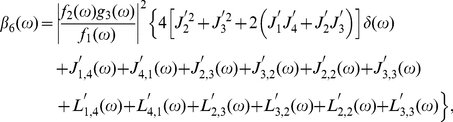
(48)

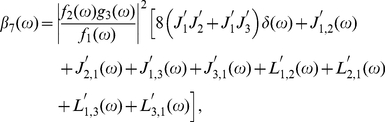
(49)

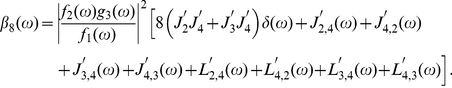
(50)With respect to
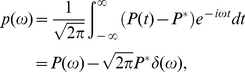
(51a)

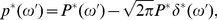
(51b)Eqs. (34) can be read as
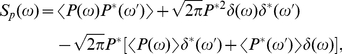
(52a)

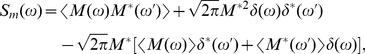
(52b)so the power spectra of 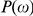 and 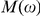 can be expanded from Eqs. (52) as
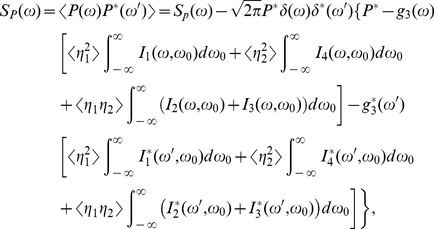
(53)

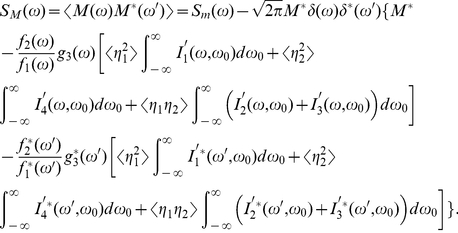
(54)


If we remove the nonlinear terms in Eqs. (21), Eqs. (34) become
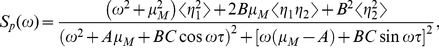
(55a)

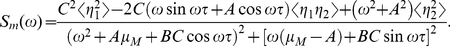
(55b)When 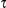 is small, an approximation can be made,
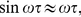
(56a)

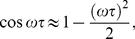
(56b)and then Eqs. (55) become
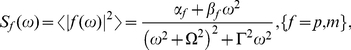
(57)where
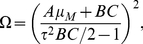
(58)

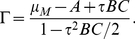
(59)For 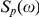, 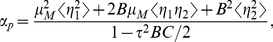
(60a)

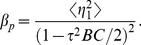
(60b)For 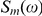,
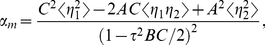
(61a)

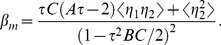
(61b)


It is worthwhile to mention that a module, which consists of two components, has been discussed recently [78]. They studied a set of coupled Langevin equations for the interacting species. It is very interesting that in the absence of delay and nonlinearity, i.e., a special case of the spectrum as 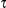 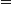 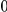 in Eqs. (55), Eqs. (34) can be reduced as
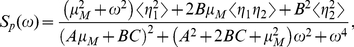
(62a)

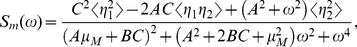
(62b)which are consistent with the results presented in the previous paper [78].

Another characteristic feature of Eqs. (34) is that when 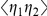 is assumed to be zero, which means that 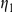 and 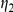 are uncorrelated, both 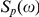 and 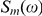 can be written as a sum of two contributions which is the so-called spectral addition rule as derived in the previous paper [78]. Even in this case, the coefficients in our results still include the effects coming from the time delay and negative feedback mechanism.

In our numerical calculation, we use the fourth-order stochastic Runge-Kutta method for integrating the chemical Langevin equations (18), and Gaussian integration method to calculate the integrations in Eqs. (34). The numerical results have shown that the correlation functions 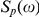 and 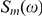 for 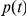 and 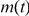 are precisely consistent between the ones with chemical Langevin equations (18) and the ones with Eqs. (34), which verifies our truncation method in Eqs. (23). The Fourier transforms of p53 and Mdm2 dynamics show that the number of the resonant peaks would increase as time delay increases, which is consistent with the experimental results [12]. The general finding of our analysis is that an increase of delay between activation and induction induces an oscillatory behavior with frequency which corresponds nearly to the delay time. The spectral analysis as well as the mutual information supports this finding. The general finding is in good agreement with our previous work [37].

Bioscience and nanoscience provide pretty examples of nonequilibrium and nonlinear dynamics in which noise can be expected to have unavoidable effects. The methods developed over years to deal with the effects in physical systems will help us to further our understanding of the mechanisms ascribed to nonlinearity and noise.

## Methods

The stochastic p53 circuit was characterized by a Monte Carlo method called the exact DSSA. Numerical integration of the equations was carried out using Matlab software.
